# Transcription suppression is mediated by the HDAC1–Sin3 complex in *Xenopus* nucleoplasmic extract

**DOI:** 10.1016/j.jbc.2022.102578

**Published:** 2022-10-08

**Authors:** Colleen E. Quaas, Baicheng Lin, David T. Long

**Affiliations:** Department of Biochemistry and Molecular Biology, Medical University of South Carolina, Charleston, South Carolina, USA

**Keywords:** bromodomain-containing protein-4, chromatin regulation, histone deacetylase 1, histone acetylation, transcription regulation, BRD, bromodomain, ChIP, chromatin immunoprecipitation, ELB, egg lysis buffer, IP, immunoprecipitation, NPE, nucleoplasmic extract, PTM, posttranslational modification

## Abstract

Modification of histones provides a dynamic mechanism to regulate chromatin structure and access to DNA. Histone acetylation, in particular, plays a prominent role in controlling the interaction between DNA, histones, and other chromatin-associated proteins. Defects in histone acetylation patterns interfere with normal gene expression and underlie a wide range of human diseases. Here, we utilize *Xenopus* egg extracts to investigate how changes in histone acetylation influence transcription of a defined gene construct. We show that inhibition of histone deacetylase 1 and 2 (HDAC1/2) specifically counteracts transcription suppression by preventing chromatin compaction and deacetylation of histone residues H4K5 and H4K8. Acetylation of these sites supports binding of the chromatin reader and transcription regulator BRD4. We also identify HDAC1 as the primary driver of transcription suppression and show that this activity is mediated through the Sin3 histone deacetylase complex. These findings highlight functional differences between HDAC1 and HDAC2, which are often considered to be functionally redundant, and provide additional molecular context for their activity.

The eukaryotic genome is bound by highly conserved proteins called histones, which are essential for the organization and compaction of genetic material within the nucleus (1). The core histone is an octamer (comprised of two copies of H2A, H2B, H3, and H4) that is wrapped by ∼147 bp of DNA to form a nucleosome (2). Nucleosomes form the basic building block of chromatin, which includes interactions between DNA, RNA, and protein. Additional linker histones and histone variants also play important roles in the formation and regulation of chromatin structure (3, 4). Cells control access to DNA by regulating the compaction and decompaction of chromatin (5). These dynamic changes in chromatin structure are critical for normal gene expression profiles and cell cycle progression (6). As a result, defects in chromatin organization underlie the development of various human diseases, including cancer (7, 8).

Chromatin structure is controlled through numerous histone posttranslational modifications (PTMs), including acetylation, ubiquitination, phosphorylation, and methylation (9, 10). The majority of histone PTMs occur at the N- and C-terminal tails of each histone subunit (11), although some modifications can also target residues within the globular and fold domains of the histone core (12). Collectively, the location, type, and number of PTMs is referred to as the histone code (13). Modified histones act as a scaffold for the recruitment of proteins with specialized reader domains that activate or repress different signaling cascades (14). In this way, the histone code influences nearly all aspects of chromatin activity, including DNA replication, transcription, and DNA repair (11).

Acetylation is one of the most important histone modifications for regulating transcription and gene expression (15). The addition and removal of acetyl groups is controlled by two super families of enzymes, histone acetyltransferases (HATs) and histone deacetylases (HDACs), respectively. Histone acetylation can influence transcription through both direct and indirect mechanisms. Acetyl groups mask the positive charge of lysine residues, thereby weakening interactions between negatively charged DNA and histones (16). Acetyl groups are also recognized by proteins that contain acetyl-lysine–binding domains called bromodomains (BRDs) (17). BRD proteins have a wide range of chromatin activities, including histone modification, chromatin remodeling, and transcription regulation (18, 19).

Both HDACs and BRD proteins have been identified as attractive targets for therapeutic intervention (20, 21, 22, 23). HDAC inhibitors have been used to treat malignancies that range from cardiovascular disease, to neurological disorders, to various cancers. Although HDAC inhibitors have found success in treating hematological cancers, they have been less effective for solid tumors (24). Inhibitors that target BRD and extraterminal (BET) proteins have also shown promise as anticancer agents, with efficacy against a broad range of cancer types (25, 26). However, our understanding of the molecular signals that connect histone modification and transcription activity remains incomplete due to the complexity of interactions involved. For example, acetylations on histone H3 lysine 9 and 27 (H3K9 and H3K27) are regarded as hallmarks of transcription activation (27, 28). In comparison, the role of specific H4 acetylations remain poorly characterized (29). Many studies that explore the effects of HDAC inhibitors also monitor changes in total acetylation, which may not reflect the dynamic change of individual modifications and their downstream effects (30, 31).

Here, we use *Xenopus* egg extracts to study the molecular signals that promote DNA compaction and transcription suppression. We show that inhibition of HDAC1/2 specifically counteracts transcription suppression by preventing chromatin compaction and protecting histone H4K5 and H4K8 from deacetylation. Acetylation of these sites supports binding of the chromatin reader BRD4, which is required for transcription. We also identify HDAC1 as the primary driver of transcription suppression and show that this activity is mediated through the Sin3 histone deacetylase complex. These results help to delineate the molecular link between HDAC1 and transcription suppression, while also providing evidence that the highly similar HDAC1 and HDAC2 enzymes have independent functions.

## Results

### Transcribed plasmids become inactivated during incubation in extract

**Figure 1:**
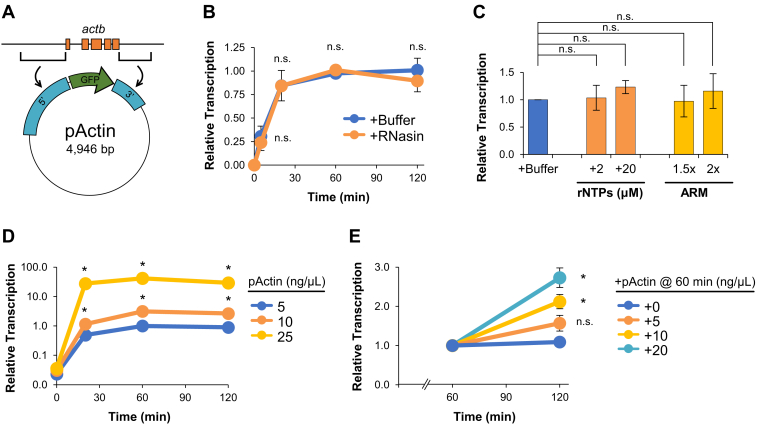
**Transcribed plasmids become inactivated during incubation in extract.***A*, schematic of pActin gene construct. *B*, pActin was incubated in NPE supplemented with buffer or an RNase inhibitor (RNasin). RNA was isolated and quantified by RT-qPCR at the times indicated (n = 2). Validation of RNasin activity is shown in Fig. S1. *C*, pActin was incubated in NPE supplemented with excess rNTPs or ARM. RNA was isolated and quantified after 120 min (n = 2). *D*, about 5 to 25 ng/μl pActin was incubated in NPE. RNA was isolated and quantified at the times indicated (n = 2). *E*, about 10 ng/μl pActin was incubated in NPE for 60 min 0 to 20 ng/μl of additional pActin was then added, and RNA was isolated and quantified after 120 min (n = 2). Student *t* test: *p*-value < 0.05 (∗), *p*-value < 0.01 (∗∗), *p*-value < 0.001 (∗∗∗), not significant (n.s.). Error bars represent ±1 SD. NPE, nucleoplasmic extract; RT-qPCR, reverse transcription quantitative PCR.

Nucleoplasmic extract (NPE) prepared from *Xenopus laevis* eggs has been shown to promote rapid histone loading and chromatinization of plasmid DNA (32, 33, 34). Recently, we found that NPE can also support robust transcription of a plasmid-borne gene construct (35, 36), providing a new tool to study how chromatin signaling influences transcription activity. In this study, we use a plasmid containing the 5′ and 3′ regulatory elements of the *Xenopus actb* gene, termed pActin (Fig. 1*A*). When pActin was incubated in NPE, we saw that accumulation of RNA transcripts produced from the *actb* promoter increased for up to 60 min and then ceased (Fig. 1*B*, *blue* trace). Supplementing reactions with an RNase inhibitor (+RNasin) did not affect transcript accumulation (Fig. 1*B*, *orange* trace), arguing that RNA was not degraded during the reaction. To test whether transcription activity was limited by consumption of available resources, we supplemented extract with additional ribonucleotides (+rNTPs) or ATP-regenerating mixture (+ARM). However, both supplements showed no significant increase in transcription compared to the buffer control (Fig. 1*C*).

Next, we compared transcription in reactions containing different amounts of pActin. Although the accumulation of transcripts increased with pActin concentration, we saw that transcription activity still ceased within 60 min (Fig. 1*D*). Thus, extracts have the capacity to support more transcription but activity is suppressed temporally. We then sought to test whether the extract itself was inactivated during incubation with pActin. Extract was incubated with 10 ng/μl of pActin for 60 min, after which time reactions were supplemented with buffer or an additional 5, 10, or 20 ng/μl of fresh pActin (Fig. 1*E*). In the buffer control, transcription did not increase between 60 and 120 min. In contrast, reactions supplemented with additional pActin showed a concentration-dependent increase in transcription. Thus, extracts retain the ability to transcribe newly added DNA. Collectively, these results argue that transcription in extract ceases due to inactivation of plasmid DNA over time.

### Inhibition of HDAC1/2 counteracts transcription suppression

**Figure 2:**
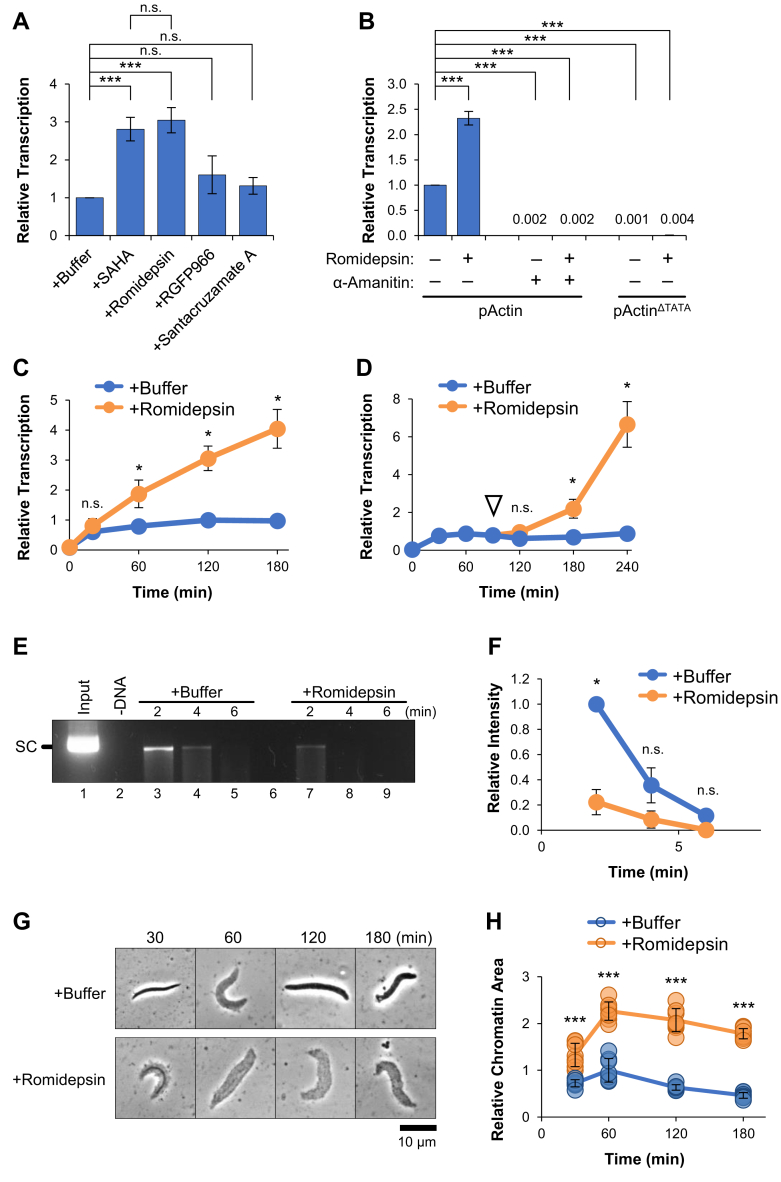
**Inhibition of HDAC1/2 counteracts transcription suppression and increases DNA accessibility.***A*, pActin was incubated in NPE supplemented with 100 μM of the indicated HDAC inhibitor. RNA was isolated and quantified after 120 min (n = 3). *B*, pActin and pActin^ΔTATA^ were incubated in NPE supplemented with 10 ng/μl α-amanitin and/or 100 μM Romidepsin, as indicated. RNA was isolated and quantified after 120 min (n = 2). *C*, pActin was incubated in NPE supplemented with buffer or 100 μM Romidepsin. RNA was isolated and quantified at the indicated time points (n = 2). *D*, pActin was incubated in NPE for 90 min. Reactions were then supplemented with either buffer or 100 μM Romidepsin. RNA was isolated and quantified at the indicated time points (n = 2). *E*, about 30 ng/μl pActin was incubated in NPE supplemented with buffer or Romidepsin for 120 min. Samples were then analyzed by the MNase cleavage assay (n = 2). Supercoiled (SC). *F*, quantification of SC plasmid intensity in (*E*) (n = 2). *G*, sperm chromatin was incubated in NPE supplemented with buffer or 100 μM Romidepsin. At the indicated time points, samples were withdrawn and visualized by phase contrast light microscopy (n = 2). *H*, quantification of two-dimensional chromatin area from (*G*). Student *t* test: *p*-value < 0.05 (∗), *p*-value < 0.01 (∗∗), *p*-value < 0.001 (∗∗∗), not significant (n.s.). Error bars represent ±1 SD. NPE, nucleoplasmic extract.

One of the primary mechanisms of transcription regulation involves acetylation and deacetylation of histones (20). Histones are highly acetylated in *Xenopus* eggs, which are thought to facilitate chromatin assembly following fertilization (37, 38). However, transcription is largely suppressed during early embryo development (39, 40). We hypothesized that plasmid transcription may be suppressed by the programmed removal of histone acetylations over time. To investigate the role of histone deacetylation in our system, pActin was incubated in extract supplemented with different HDAC inhibitors. Highly selective HDAC inhibitors have been developed for clinical use and have well-established substrate profiles (41). We first titrated each inhibitor into extract to determine the most effective concentrations (Fig. S2*A*), which are compared in Figure 2*A*. Notably, NPE contains a highly concentrated fraction of nuclear proteins and typically requires high doses for effective inhibition (42, 43, 44, 45). In the presence of a global class I, II, and IV HDAC inhibitor called SAHA (Suberoylanilide hydroxamic acid) (46), transcription increased ∼3-fold compared to the buffer control, indicating that transcription was limited by HDAC activity. We then sought to identify the specific HDACs involved. We focused on class I enzymes (HDAC1, HDAC2, HDAC3, and HDAC8), which are generally localized to the nucleus and classified as transcriptional repressors (47). We found that reactions supplemented with Santacruzamate A (HDAC2 inhibitor) or RGFP966 (HDAC3 inhibitor) did not significantly increase in transcription (Fig. 2*A*) (48, 49). In contrast, Romidepsin (HDAC1/2 inhibitor) (50) increased transcription ∼3-fold. Similar results were also obtained with different gene constructs (Fig. S2, *B* and *C*), arguing that Romidepsin’s effect was not dependent on DNA sequence. To rule out nonspecific accumulation of RNA in the presence of Romidepsin, reactions were first supplemented with the RNA polymerase II (RNAPII) inhibitor ⍺-amanitin. RNAPII inhibition completely blocked transcription in both the buffer- and Romidepsin-treated reactions (Fig. 2*B*). Reactions were then performed using pActin plasmid that lacked a TATA box (pActin^ΔTATA^). Again, transcription was blocked in both buffer- and Romidepsin-treated reactions (Fig. 2*B*). These results indicate that the effects of Romidepsin were dependent on both RNAPII and a functional *actb* promoter.

To further investigate the consequences of HDAC1/2 inhibition, we first analyzed how Romidepsin affected transcription of pActin over time. Up to 30 min, transcription was similar in reactions treated with buffer or Romidepsin (Fig. 2*C*). However, in the presence of Romidepsin, transcription was not suppressed and remained active throughout the reaction (up to 180 min). We then tested whether Romidepsin could restore transcription of pActin after it had been inactivated. pActin was incubated in extract for 90 min. The reaction was then split and supplemented with buffer or Romidepsin. While the buffer control remained inactive, transcriptional activity was restored after adding Romidepsin (Fig. 2*D*). Taken together, these data show that inhibition of HDAC1 and HDAC2 can prevent or counteract transcription suppression.

### Romidepsin increases DNA accessibility

Histone deacetylation promotes broad changes in chromatin structure that are associated with gene silencing (51). To investigate how HDAC1/2 inhibition affected DNA accessibility in NPE, we tested the sensitivity of pActin to cleavage by micrococcal nuclease (MNase), which exhibits both exonuclease and endonuclease activity against exposed dsDNA. Reactions were supplemented with buffer or Romidepsin for 120 min. Samples were then withdrawn and treated with a limiting amount of MNase for increasing amounts of time and resolved by agarose gel electrophoresis. In the presence of Romidepsin, pActin was cleaved more quickly than the buffer control (Fig. 2, *E* and *F*), indicating that plasmid DNA was more accessible to MNase activity.

To directly visualize the effects of HDAC1/2 inhibition on chromatin structure, we substituted pActin (4.9 kb) with the much larger *Xenopus laevis* sperm chromatin (∼3.1 Gb) (52). Reactions were supplemented with buffer or Romidepsin and samples were withdrawn at various times for visualization by light microscopy. Purified sperm chromatin is bound by protamines that keep individual chromosomes tightly packed. When added to extract, protamines are rapidly replaced by histones that control the state of chromatin compaction (53). In the buffer control, chromatin area increased from 30 to 60 min and then decreased thereafter (Fig. 2, *G* and *H*). In the presence of Romidepsin, there was an increase in chromatin area that persisted up to 180 min, indicating a failure to compact chromatin after histone exchange (Fig. 2, *G* and *H*). In both the buffer- and Romidepsin-treated reactions, the timing of initial chromatin decompaction coincided with active transcription of pActin (Fig. 2*C*), suggesting that both processes are linked. Collectively, these results argue that HDAC1/2 inhibition stimulates transcription of pActin, at least in part, by promoting chromatin decompaction.

### Romidepsin stimulates BRD4-dependent transcription

**Figure 3:**
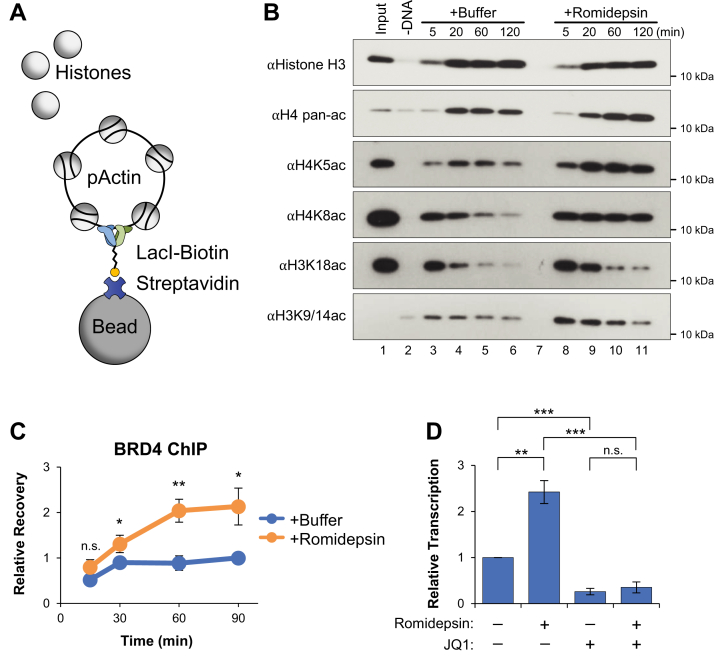
**Romidepsin stimulates BRD4-dependent transcription.***A*, plasmid pull-down schematic. *B*, pActin was incubated in NPE supplemented with buffer or 100 μM Romidepsin. At the indicated time points, DNA-bound proteins were isolated by plasmid pull down and visualized by Western blot with the indicated antibodies (n = 2). Input represents 3% of total reaction sample. Acetylation of H4K5 and H4K8 in total reaction samples is shown in Fig. S3*A*. *C*, pActin was incubated in NPE supplemented with buffer or 100 μM Romidepsin. Samples were withdrawn at the indicated time points and binding of BRD4 to the *actb* promoter region was analyzed by ChIP (n = 3). *D*, pActin was incubated in NPE supplemented with Romidepsin and/or JQ1, as indicated. RNA was isolated and quantified after 120 min (n = 2). Student *t* test: *p*-value < 0.05 (∗), *p*-value < 0.01 (∗∗), *p*-value < 0.001 (∗∗∗), not significant (n.s.). Error bars represent ±1 SD. BRD, bromodomain; NPE, nucleoplasmic extract.

In cells, Romidepsin treatment has been shown to induce widespread changes in global acetylation levels (54). To identify the specific histone acetylations controlled by HDAC1/2, DNA-bound histones were isolated by LacI plasmid pull down (Fig. 3*A*) and visualized by Western blot. Initially, histone loading occurred rapidly after the addition of pActin to extract, as shown by the accumulation of DNA-bound histone H3 within 20 min (Fig. 3*B*). Using a pan-acetyl antibody, we saw that the overall level of histone H4 acetylation remained stable in both buffer- and Romidepsin-treated reactions. However, the accumulation of specific H3 and H4 acetylations was more dynamic. In the buffer control, we saw that H4K5ac, H4K8ac, H3K18ac, and H3K9/14ac all decreased over time after initial histone loading (Fig. 3*B*, lanes 3–6), identifying them as putative targets for deacetylation by HDAC1/2. Additional histone acetylations were blotted but not detected (Fig. S3*B*). When treated with Romidepsin, two residues were protected from deacetylation by HDAC1/2: H4K5ac and H4K8ac (Fig. 3*B*, lanes 8–11). Although the level of H3K18ac and H3K9/14ac initially increased with Romidepsin treatment, the loss of signal over time argues that they were not responsible for the prolonged increase in transcription seen in Figure 2*C*. Notably, the chromatin reader BRD4 is preferentially recruited to histones by acetylation of H4K5, H4K8, and H4K12 (55, 56), suggesting that Romidepsin may regulate transcription through BRD4 binding (57).

We then sought to confirm that transcriptionally active plasmids were linked to the histone modifications observed in Figure 3*B*. RNAPII was immunoprecipitated from buffer- or Romidepsin-treated reactions to isolate transcriptionally active plasmids. The recovered DNA-bound histones were then visualized by Western blot. We saw that RNAPII-bound plasmids recovered H4K8ac and that acetylation increased in the presence of Romidepsin (Fig. S3*C*).

To test whether Romidepsin treatment impacts BRD4 binding, its recruitment to the *actb* promoter was analyzed by chromatin immunoprecipitation (IP) (ChIP). Compared to the buffer control, reactions supplemented with Romidepsin showed a roughly 2-fold increase in BRD4 accumulation (Fig. 3*C*). Notably, binding of BRD4 in the buffer control did not diminish at later times, indicating that BRD4 binding may be necessary but not sufficient for transcription activity. To test whether the increased transcription observed with Romidepsin treatment was dependent on BRD4 activity, reactions were supplemented with buffer, Romidepsin, JQ1 (a highly selective BET inhibitor that blocks BRD4 binding) (58), or a combination of both drugs. We saw that BRD4 inhibition blocked transcription in both the buffer- and Romidepsin-treated reactions (Fig. 3*D*). These data indicate that HDAC1/2 suppresses transcription, in part, through the direct regulation of BRD4 binding.

**Figure 4:**
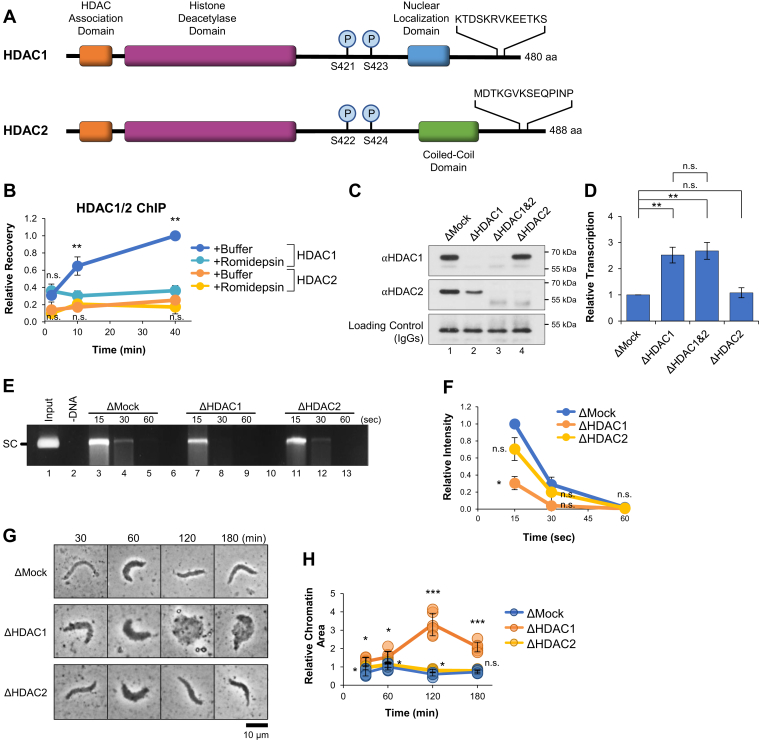
**HDAC1 drives transcription suppression.***A*, schematic representation of HDAC1 and HDAC2. Unique amino acid sequences used to generate peptide antigens are shown at the C terminus for each protein. *B*, pActin was incubated in NPE supplemented with buffer or Romidepsin. Samples were withdrawn at the indicated time points and binding of HDAC1 and HDAC2 to the *actb* promoter region was analyzed by ChIP (n = 3). Significance shown for comparison between +Buffer and +Romidepsin reactions. *C*, NPE was immunodepleted using preimmune (ΔMock), HDAC1 (ΔHDAC1), HDAC2 (ΔHDAC2), or a combination HDAC1 and 2 (ΔHDAC1&2) antibodies. Depleted extracts were analyzed by Western blot with the indicated antibodies. *D*, pActin was incubated in depleted extracts from (*C*). RNA was isolated and quantified after 120 min (n = 3). *E*, pActin was incubated in mock-, HDAC1-, or HDAC2-depleted extract for 120 min. Samples were then analyzed by the MNase cleavage assay (n = 2). Supercoiled (SC). *F*, quantification of SC plasmid intensity in (*E*). *G*, sperm chromatin was incubated in mock-, HDAC1-, or HDAC2-depleted extracts. At the indicated time points, samples were withdrawn and visualized by phase contrast light microscopy (n = 2). *H*, quantification of two-dimensional chromatin area in (*G*). Student *t* test: *p*-value < 0.05 (∗), *p*-value < 0.01 (∗∗), *p*-value < 0.001 (∗∗∗), not significant (n.s.). Error bars represent ±1 SD. ChIP, chromatin immunoprecipitation; NPE, nucleoplasmic extract.

### HDAC1 drives transcription suppression

HDAC1 and HDAC2 are considered to be functionally redundant in most cases (59) and are found interchangeably in different deacetylase complexes (60). However, growing evidence argues that the proteins have individual functions in some contexts (61, 62, 63). HDAC1 and HDAC2 share high homology (80% in humans and 85% in *Xenopus laevis*) and have nearly identical catalytic domains (Fig. 4*A*) (59). As a result, Romidepsin effectively targets the catalytic site of each protein and cannot discriminate between their activities (50). Therefore, to investigate the individual functions of HDAC1 and HDAC2, we developed antibodies that specifically recognize each *Xenopus* protein (Fig. S4). We compared the recruitment of HDAC1 and HDAC2 to the *actb* promoter by ChIP in reactions supplemented with buffer or Romidepsin. HDAC1 binding increased over time in the buffer control but was blocked in the presence of Romidepsin (Fig. 4*B*, *blue* and *cyan* traces, respectively). Although Romidepsin is not predicted to interfere with HDAC1/2 binding directly, its effect on protein localization is not well characterized (50, 64). We hypothesize that binding to the promoter was reduced due to nucleosome remodeling associated with increased transcriptional activity (65, 66). In contrast to HDAC1, recovery of HDAC2 remained low in both the buffer- and Romidepsin-treated reactions (Fig. 4*B*, *orange* and *yellow* traces, respectively). Thus, HDAC1 and HDAC2 exhibit differences in DNA-binding activity, suggesting that they also play different roles during transcription suppression.

To further investigate the relative roles of HDAC1 and HDAC2 in transcription suppression, extract was immunodepleted using mock (nonspecific IgGs), HDAC1, HDAC2, or a combination of HDAC1 and HDAC2 antibodies. Notably, depletion of HDAC1 codepleted ∼50% of HDAC2 (Fig. 4*C*, lane 2), suggesting that the majority of HDAC2 was found in mixed complexes with HDAC1. In contrast, depletion of HDAC2 had little or no effect on the level of HDAC1 (Fig. 4*C*, lane 4), suggesting that the majority of HDAC1 was found in HDAC1-only complexes. Depletion of HDAC1 or both HDAC1 and HDAC2 increased transcription ∼2.5-fold compared to the mock-depleted control (Fig. 4*D*). In contrast, depletion of HDAC2 alone had no effect on transcription activity. These results argue that the level of HDAC2 does not significantly influence transcription and that suppression is driven by HDAC1. They also provide additional evidence that HDAC1 and HDAC2 have distinct functions in transcription regulation.

We then investigated how loss of HDAC1 or HDAC2 affected DNA accessibility. We first incubated pActin in mock-, HDAC1-, or HDAC2-depleted extract. Samples were withdrawn after 120 min, treated with MNase, and resolved by agarose gel electrophoresis. We saw that pActin was more accessible to MNase cleavage in the HDAC1-depleted reaction than in the mock- or HDAC2-depleted reactions, which were cleaved with similar kinetics (Fig. 4, *E* and *F*). We then incubated sperm chromatin in mock-, HDAC1-, or HDAC2-depleted extract and samples were visualized by light microscopy. We saw that chromatin area increased dramatically in HDAC1-depleted reactions, while both mock- and HDAC2-depleted reactions showed normal patterns of chromatin decompaction and compaction (Fig. 4, *G* and *H*). We speculate that HDAC1 depletion is more severe than treatment with Romidepsin (Fig. 2*G*) due to removal of the entire complex and its associated functions (67, 68). Together, these results argue that HDAC1 controls DNA accessibility and chromatin compaction, which correlate with transcription activity.

### HDAC1/2 occupancy in deacetylase complexes

**Figure 5:**
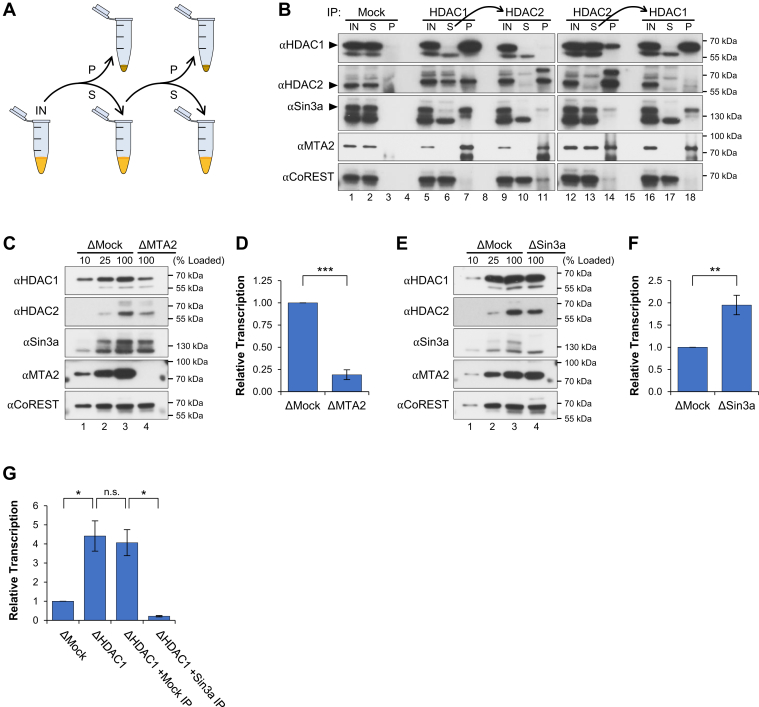
**The Sin3 deacetylase complex promotes transcription suppression.***A*, sequential IP schematic. *B*, mock, HDAC1, or HDAC2 IPs were performed in NPE. The supernatants from HDAC1 or HDAC2 IPs were then used for a second round of IPs using the converse antibody. Isolated proteins were then analyzed by Western blot with the indicated antibodies (n = 3). 10% of total reaction sample (IN), supernatant (S), pellet (P). *C*, NPE was immunodepleted using preimmune (ΔMock) or MTA2 (ΔMTA2) antibodies. Depleted extracts were analyzed by Western blot using the indicated antibodies. *D*, pActin was incubated in mock- or MTA2-depleted extract. RNA was isolated and quantified after 120 min (n = 2). *E*, NPE was immunodepleted using preimmune (ΔMock) or Sin3a (ΔSin3a) antibodies. Depleted extracts were analyzed by Western blot using the indicated antibodies. *F*, pActin was incubated in mock- or Sin3a-depleted extract. RNA was isolated and quantified after 120 min (n = 2). *G*, NPE was immunodepleted using preimmune (ΔMock) or HDAC1 (ΔHDAC1) antibodies. HDAC1-depleted extract was then supplemented with immunoprecipitated proteins recovered by preimmune (+Mock IP) or Sin3a (+Sin3a IP) IP. pActin was incubated in each extract and RNA was isolated and quantified after 120 min (n = 2). Student *t* test: *p*-value < 0.05 (∗), *p*-value < 0.01 (∗∗), *p*-value < 0.001 (∗∗∗), not significant (n.s.). Error bars represent ±1 SD. IP, immunoprecipitation; NPE, nucleoplasmic extract.

HDAC1 and HDAC2 are found in at least three major macromolecular complexes, including Sin3, NuRD, and CoREST, which have been shown to play distinct roles in transcription regulation (69, 70, 71). Each complex carries either two copies of HDAC1, two copies of HDAC2, or one of each enzyme. Complex formation is essential for HDAC1 and HDAC2 activity, as it regulates their localization and interaction with target substrates (68, 72). The variable nature of HDAC1/2 occupancy within different complexes has complicated efforts to elucidate the individual functions of each enzyme (73). To determine the relative occupancy of HDAC1 and HDAC2 in different complexes, we used sequential IPs to isolate HDAC1- or HDAC2-only complexes (Fig. 5*A*). First, IPs were performed with mock, HDAC1, or HDAC2 antibodies. Next, supernatant from the HDAC1 or HDAC2 IPs was used for a second round of IPs with the converse antibody. Supernatant and pellet samples from each IP were then analyzed by Western blot with antibodies that recognize subunits from each deacetylase complex (Sin3a for Sin3, MTA2 for NuRD, and CoREST for CoREST). The HDAC1 IP removed HDAC1 and ∼50% of HDAC2 (Fig. 5*B*, lanes 6 and 7). IP of the remaining HDAC2-only complexes recovered MTA2, CoREST, and a small amount of Sin3a (Fig. 5*B*, lane 11). The HDAC2 IP removed HDAC2 and a small fraction of HDAC1 (Fig. 5*B*, lanes 13 and 14). IP of the remaining HDAC1-only complexes recovered Sin3a and MTA2 but not CoREST (Fig. 5*B*, lane 18). Notably, the amount of Sin3a, MTA2, and CoREST recovered in the first and second round IPs was similar for both the HDAC1 and HDAC2 sequential IPs (Fig. 5*B*, compare lanes 7 and 18, or 11 and 14), indicating that the amount of mixed HDAC1/2 complexes was relatively low compared to the HDAC1- and HDAC2-only complexes.

Given that HDAC1 was primarily associated with the Sin3 and NuRD complexes, we then sought to determine the role that each complex plays in transcription suppression. We first immunodepleted MTA2 from extract, which was specific for the NuRD complex and did not codeplete Sin3a or CoREST (Fig. 5*C*, lanes 3 and 4). MTA2 depletion did codeplete the majority of both HDAC1 and HDAC2, indicating that a sizeable fraction of each enzyme is in the NuRD complex. When pActin was incubated in mock- or MTA2-depleted extracts, we saw that loss of MTA2 severely impaired transcription (Fig. 5*D*). The NuRD complex has been implicated in stimulating transcription through upregulation of super-enhancer elements *via* the chromodomain helicase DNA-binding protein 4 (CHD4) subunit. Importantly, the NuRD complex has also been shown to interact with BRD4 (74). Depletion of MTA2 did not codeplete BRD4 from extract (Fig. S5*A*) but did reduce binding of BRD4 to the *actb* promoter by ∼40% (Fig. S5*B*), arguing that the NuRD complex plays a role in stimulating BRD4-dependent transcription. However, we cannot rule out the possibility that the NuRD complex also contributes to transcription suppression independently of its role in transcription activation.

We then immunodepleted Sin3a from extract, which was specific for the Sin3 complex and did not codeplete MTA2 or CoREST (Fig. 5*E*, lanes 3 and 4). Sin3a depletion did not substantially affect the level of HDAC1 or HDAC2, suggesting that only a minor fraction of total HDAC1 is in the Sin3 complex. However, when pActin was incubated in mock- or Sin3a-depleted extract, we saw that loss of Sin3a increased transcription ∼2-fold (Fig. 5*F*), similar to the effect of HDAC1 depletion (Fig. 4*D*) and Romidepsin treatment (Fig. 2*A*). To further investigate the link between HDAC1 and the Sin3 complex, we performed IPs using mock or Sin3a antibodies. The bead-bound proteins were then added to HDAC1-depleted extracts to test whether the endogenous Sin3 complex could rescue HDAC1-mediated transcription suppression. When beads from the mock IP were added to HDAC1-depleted extract, it had no effect on transcription (Fig. 5*G*). In contrast, beads from the Sin3a IP suppressed transcription back below the level seen in mock-depleted reactions. Together, these results argue that HDAC1’s role in transcription suppression is mediated through the Sin3 complex.

## Discussion

In this report, we investigate the dynamic regulation of transcription activity in NPE. We found that transcription was temporally suppressed, not due to a lack of resources within extract but due to inactivation of plasmid DNA (Fig. 1). Suppression of transcription could be prevented or counteracted by treatment with Romidepsin, specifically implicating HDAC1/2 (Fig. 2, *A*–*D*). In cells, Romidepsin treatment promotes widespread decompaction of chromatin (75) which is consistent with the effects observed in extract (Fig. 2, *E*–*H*). These results demonstrate that NPE recapitulates mechanisms of chromatin compaction mediated by histone acetylation, highlighting the system’s potential for *in vitro* study of chromatin dynamics in the context of epigenetic modification.

We also identify specific histone acetylations targeted by HDAC1/2 and connect these modifications to the chromatin reader and transcription regulator BRD4. We show that HDAC1/2 inhibition protects H4K5 and H4K8 from deacetylation (Fig. 3*B*). Although HDAC3 has been shown to target H4K5 and H4K8 (76, 77), direct regulation of these marks by HDAC1/2 has not been clearly established (78, 79). We then show that HDAC1/2 inhibition enhanced BRD4 binding, which was required for elevated transcription (Fig. 3, *C* and *D*). These results delineate a direct role for HDAC1/2 in suppressing transcription through regulation of BRD4 binding (80, 81). Thus, HDAC1/2 controls transcription both indirectly through changes in chromatin structure and more directly through regulated protein recruitment.

HDAC1 and HDAC2 are often considered to be functionally redundant due to the similarity of their catalytic domains and the complexes they occupy. Here, we provide new insight on the molecular functions of HDAC1, further establishing the individual roles of HDAC1 *versus* HDAC2. We show that HDAC1 and HDAC2 exhibit differences in DNA binding (Fig. 4*B*), play distinct roles in chromatin compaction (Fig. 4, *E*–*H*), and occupy different deacetylase complexes (Fig. 5*B*). We further identify the HDAC1–Sin3 complex as the primary mediator of transcription suppression (Fig. 5, *E*–*G*), providing molecular context for HDAC1’s role in transcription suppression.

Currently, the HDAC1/2 inhibitor Romidepsin is one of five US Food and Drug Administration (FDA)–approved HDAC inhibitors (50). Although the clinical effectiveness of Romidepsin has been limited primarily to hematological cancers, an increasing number of combinational therapies are now being evaluated in clinical trials for various diseases, including relapsed refractory T-cell lymphoma (NCT03770000) (https://clinicaltrials.gov/ct2/show/NCT03770000), pancreatic cancer (NCT04257448) (https://clinicaltrials.gov/ct2/show/NCT04257448), and HIV infection (NCT03619278) (https://clinicaltrials.gov/ct2/show/NCT03619278). These studies highlight the broad role that chromatin signaling plays in human health and demonstrate the potential impact that new molecular insights hold for treatment of numerous human malignancies.

## Experimental procedures

### Reactions in *Xenopus* egg extract

*Xenopus laevis* were cared for and used according to approved IACUC and AAALAC protocols. *Xenopus* egg extracts and sperm chromatin were prepared as described previously (82). In brief, NPE was formed by incubating demembranated sperm chromatin in crude egg extract to form nuclei. Nuclei were isolated by centrifugation and then fractionated by ultracentrifugation to remove lipids and chromatin. The pActin, pActin^ΔTATA^, pCMV, and pBRCA1 plasmids were constructed as described previously (35). For all reactions, extracts were supplemented with 1 mM DTT and ATP regenerating mix (6.5 mM phosphocreatine, 0.65 mM ATP, and 1.6 μg/ml creatine phosphokinase). Prior to the addition of DNA (T = 0 min), extracts were incubated at 21 °C (room temperature [RT]) for 10 min. Unless otherwise indicated, reactions were supplemented with 10 ng/μl plasmid DNA or 1250 demembranated sperm chromatin/μl. Where indicated, reactions were also supplemented with 10 to 125 μM HDAC inhibitors: SAHA (Cayman 10009929), Romidepsin (Selleckchecm S3020), RGFP966 (Selleckchem S7229), Santacruzamate A (Selleckchem S7595), 300 μM JQ1 (Sigma SML1524), 20 ng/μl ⍺-amanitin, 2 to 20 μM rNTPs, 0.5 ng/μl RNase A (Fisher), and/or 4 units/μl RNasin (Promega). All experiments were performed with at least two biological replicates and representative or average data are shown.

### Reverse transcription quantitative PCR

RNA was isolated from extract using the EZNA RNA Purification kit (Omega BioTek) and complementary DNA was then generated using the QuantiTect Reverse Transcription kit (Qiagen). Samples were then analyzed by reverse transcription quantitative PCR with the following primer pairs:

*actb* Promoter (+10 to +153 bp from the transcription start site):

Forward: CCCGCATAGAAAGGAGACA

Reverse: GCCAGAACATAGACATTAAGAAGG

CMV Promoter (+52 to +198 bp from the transcription start site):

Forward: AGCTGGACGGCGACGTAAAC

Reverse: AGGTCAGGGTGGTCACGAGG

18S Control:

Forward: GACCGGCGCAAGACGAACCA

Reverse: TGCTCGGCGGGTCATGGGAA

Results were normalized using endogenous 18S rRNA present within the extract.

### Plasmid pull down

DNA-bound proteins were isolated from extract as described previously (83). Briefly, 8 μl reaction samples were withdrawn at the indicated time points and incubated with LacI-bound magnetic beads (Dynabeads M-280; Invitrogen) suspended in 40 μl of pull-down buffer (10 mM Hepes [pH 7.7], 50 mM KCl, 2.5 mM MgCl_2_, 250 mM sucrose, 0.25 mg/ml bovine serum albumin, and 0.02% Tween 20) for 20 min at 4 °C. Beads were washed three times with wash buffer (10 mM Hepes [pH 7.7], 50 mM KCl, 2.5 mM MgCl_2_, 0.25 mg/ml bovine serum albumin, and 0.03% Tween 20), dried, and eluted using 2× SDS sample buffer (100 mM Tris–HCl [pH 6.8], 4% SDS, 0.2% bromophenol blue, 20% glycerol, and 200 mM β-mercaptoethanol). Isolated proteins were then then resolved by SDS-PAGE and visualized by Western blot.

### Antibodies, immunodepletion, and IP

Commercial antibodies were used to detect Histone H3 (ThermoFisher PA5-16183), H4K5ac (Abclonal A15233), H4K8ac (Abclonal A7258), H4K16ac (Abclonal A5280), H3K27ac (Abclonal A7253), MTA2 (Novus Biologicals NB100-56483SS), CoREST (Millipore 07-455), H2AK5ac (Thermofisher 720070), H2BK20ac (Millipore Sigma 07-347), H3K9/14ac (ThermoFisher 49-1010), H3K23ac (Cell Signaling 8848), and RNAPII (Bethyl Laboratories A300-653A). The *Xenopus* Sin3a antibody was a gift from Dr Peter L. Jones (University of Nevada, Reno). Antibodies for *Xenopus* HDAC1, HDAC2, and BRD4 were produced by New England Peptide (NEP) using the following antigen sequences: HDAC1, KTDSKRVKEETKS; HDAC2, MDTKGVKSEQPINP; and BRD4, NFQSELMEIFEQNLFS. Immunodepletion was performed as described previously (35). Briefly, to immunodeplete HDAC1, HDAC2, or Sin3a, 48 μl of serum was conjugated to 4 μl of Protein-A Sepharose beads (VWR) and incubated with 10 μl of NPE at 4 °C for 1 h over two rounds. For MTA2, 2 μg of antibody was conjugated to 4 μl of Protein-A Sepharose beads and incubated with 10 μl of NPE at 4 °C for 1 h over two rounds. For mock-depleted controls, an identical immunodepletion was performed with preimmune serum or an equivalent concentration of purified preimmune antibody. For IPs, 5 μl of serum or 2 μg of purified antibodies were conjugated to 5 μl of Protein-A Sepharose beads. Beads were incubated at 4 °C for 120 min with either NPE or reaction samples that were diluted 5× in egg lysis buffer (ELB; 10 mM Hepes–KOH pH 7.7, 2.5 mM MgCl_2_, 50 mM KCl, and 250 mM sucrose) and then washed three times with ELB. For rescue experiments, beads were suspended in depleted extract and then incubated at 21 °C for 120 min with rotation. For Western blotting, beads were eluted with 2× SDS sample buffer and isolated proteins were resolved by SDS-PAGE.

### Agarose gel electrophoresis

At the indicated time points, reaction samples were withdrawn and added to 5 μl reaction stop buffer (3.6% SDS, 18 mM EDTA, 90 mM Tris–HCl pH 8, 90 mg/ml Ficoll, and 3.6 mg/ml bromophenol blue). Samples were incubated with 10 μg/μl proteinase K at RT for 16 h, resolved by 0.8% agarose gel electrophoresis, and visualized using SYBR Gold Stain (Thermo Scientific S11494).

### ChIP

ChIP was performed as described previously (83). Briefly, reaction samples were crosslinked in ELB containing 1% formaldehyde. Crosslinking was quenched with 125 mM glycine and then excess formaldehyde was removed using a Micro Bio-Spin 6 chromatography column (Bio-Rad). Samples were then sonicated (Diagenode Bioruptor UCD-600 TS) and immunoprecipitated with the indicated antibodies coupled to Protein-A Sepharose beads. Samples were decrosslinked and DNA was isolated *via* phenol/chloroform extraction and ethanol precipitation. Recovered DNA was then analyzed by reverse transcription quantitative PCR using primers that amplify a region surrounding the *actb* promoter (−120 to +71 bp from the transcription start site):

Forward: CCTCCTTCGTCCGCAGTTCC

Reverse: GCTGGCGAACCGCTACTTGC

Recovery of each sample was graphed as the percent of total input sample.

### Micrococcal nuclease cleavage assay

The MNase cleavage assay was performed as previously described (35). Briefly, reaction samples were combined with 1× MNase reaction buffer (NEB). Micrococcal nuclease was added at a final concentration of 500 units/μl, which promotes limited cleavage of plasmid DNA at reaction concentrations. Samples were then incubated at RT for the indicated time. After cleavage, samples were added to MNase STOP buffer (160 mM EDTA, 6.8% SDS) and incubated with 10 μg/μl proteinase K (Thermo fisher 501003312) for 60 min at 37 °C. Samples were then resolved by 1.5% agarose gel and visualized using SYBR Gold Stain.

## Data availability

All data are contained within the article and supporting information.

## Supporting information

This article contains supporting information.

## Conflict of interest

The authors declare that they have no conflicts of interest with the contents of this article.
