# Supplementary material for: Transcription suppression is mediated by the HDAC1–Sin3 complex in Xenopus nucleoplasmic extract
Source: J Biol Chem. 2022 Oct 8;298(11):102578. doi: 10.1016/j.jbc.2022.102578 (PMC9650048; doi:10.1016/j.jbc.2022.102578)
Supplement: Supplemental Figures S1–S5 [file mmc1.docx]

**Supporting Information for:**

**Transcription suppression is mediated by the HDAC1-Sin3 complex**

**in *Xenopus* nucleoplasmic extract**

Colleen E. Quaas^1^, Baicheng Lin^1^, and David T. Long^1*^

^1^Department of Biochemistry and Molecular Biology, Medical University of South Carolina

*To whom correspondence should be addressed: longdt@musc.edu

**
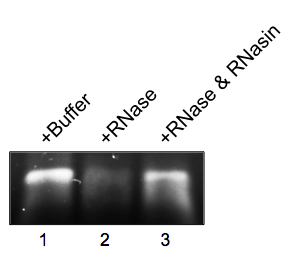
**

**Figure S1. RNA degradation and protection.** To validate the use of RNasin in extract, NPE was supplemented with buffer, RNase, or a combination of RNase and RNasin and incubated at RT for 20 minutes. Samples were then resolved by agarose gel electrophoresis and the presence of endogenous RNA was visualized with SYBR Gold stain (n=2).

**
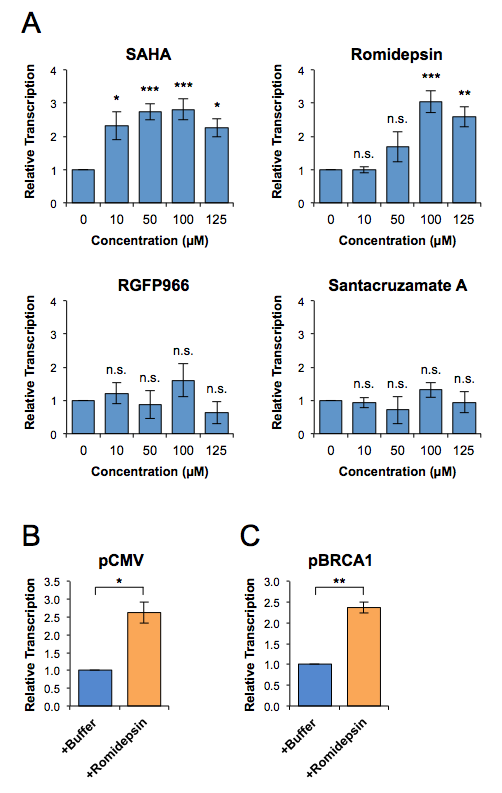
**

**Figure S2.** (**A**) pActin was incubated in NPE supplemented with buffer or the indicated amount of SAHA, Romidepsin, RGFP966, or Santacruzamate A. RNA was isolated and quantified after 120 minutes (n=3). Error bars represent +/- one standard deviation. (**B-C**) pCMV *(B)* or pBRCA1 *(C)* was incubated in NPE supplemented with buffer or 100 µM Romidepsin. RNA was isolated and quantified after 120 minutes (n=2). Student t-test: p-value < 0.05 (*), p-value < 0.01 (**), p-value < 0.001 (***), not significant (n.s.). Error bars represent +/- one standard deviation.

**
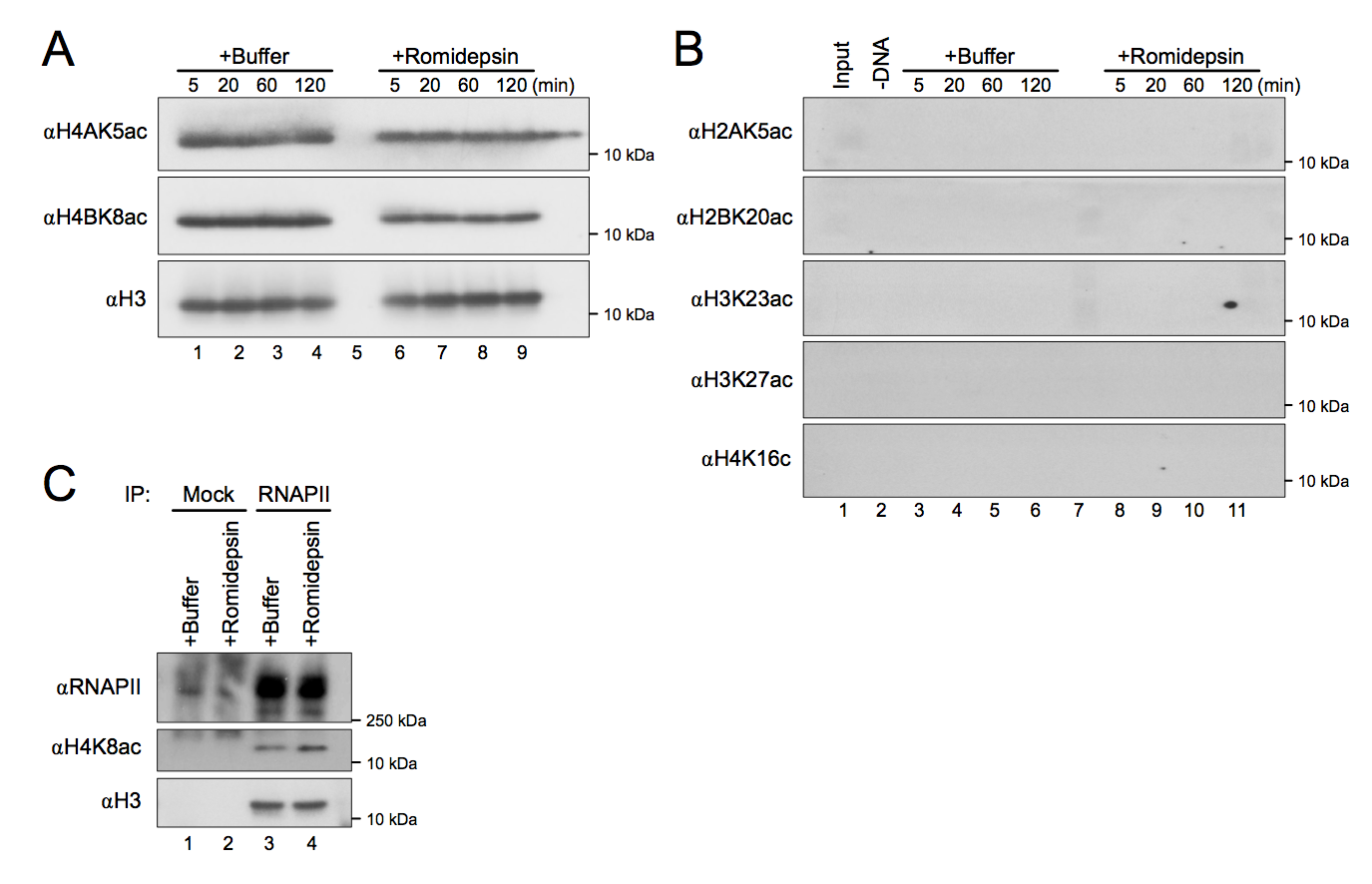
**

**Figure S3. Total histone acetylation and undetected modifications.** (**A**) Total reaction samples from Figure 3B were analyzed by Western blot with the indicated antibodies (n=2). (**B**) pActin was incubated in NPE supplemented with buffer or 100 µM Romidepsin. At the indicated time points, DNA-bound proteins were isolated by plasmid pull-down and visualized by Western blot with the indicated antibodies (n=3). Input represents 3% of total reaction sample. Note that lack of signal may represent low histone modification or low activity by the indicated antibodies. (**C**) pActin was incubated in NPE supplemented with buffer or Romidepsin for 60 minutes. Samples were withdrawn for IP with pre-immune (Mock) or RNAPII antibodies and isolated proteins were analyzed by Western blot with the indicated antibodies (n=2).


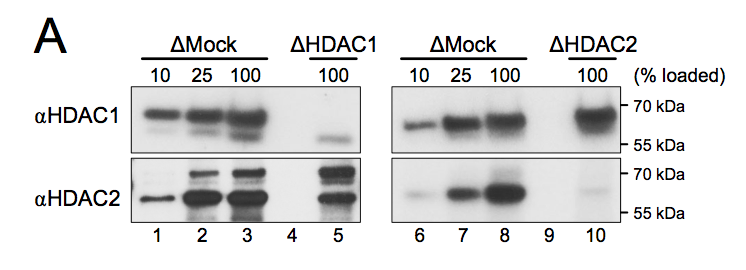


**Figure S4. Characterization of *Xenopus laevis* HDAC1 and HDAC2 antibodies.** (**A**) NPE was immunodepleted using pre-immune (∆Mock), HDAC1 (∆HDAC1), or HDAC2 (∆HDAC2) antibodies. Different amounts of depleted extract were then loaded to analyze the extent of HDAC1 and HDAC2 depletion in each extract by Western blot (n=3).


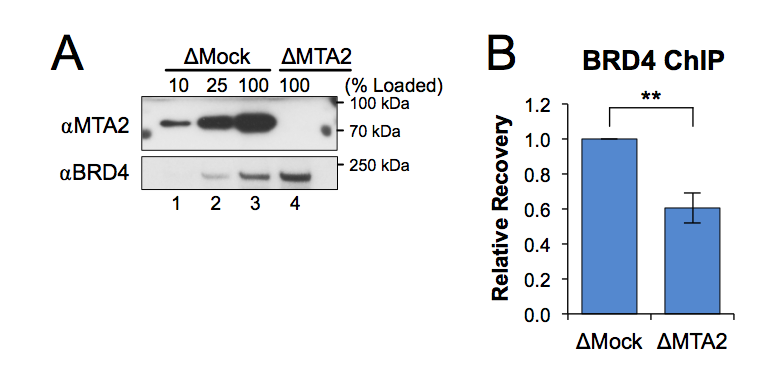


**Figure S5. MTA2 depletion reduces BRD4 binding.** (**A**) NPE was immunodepleted using pre-immune (∆Mock) or MTA2 (∆MTA2) antibodies. Depleted extracts were analyzed by Western blot using the indicated antibodies (n=2). MTA2 blot is reproduced from Figure 5C for comparison. (**B**) pActin was incubated in mock- or MTA2-depleted extract. Samples were withdrawn after 90 minutes and binding of BRD4 to the *actb* promoter region was analyzed by ChIP (n=2). Student t-test: p-value < 0.05 (*), p-value < 0.01 (**), p-value < 0.001 (***), not significant (n.s.). Error bars represent +/- one standard deviation.
